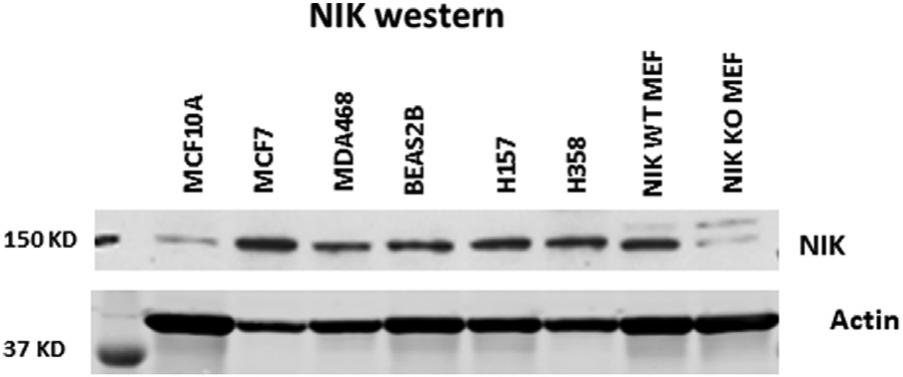# Correction: A Novel NF-κB-inducing Kinase-MAPK Signaling Pathway Up-regulates NF-κB Activity in Melanoma Cells

**DOI:** 10.1016/j.jbc.2022.102315

**Published:** 2022-08-12

**Authors:** Punita Dhawan, Ann Richmond

In the original version of Figure 3*A*(ii) the first three lanes for the actin loading control were mistakenly duplicated in the last three lanes of the image panel. A representative data from several repeated experiments is now provided in corrected Figure 3*A*(ii). The revised figure shows results obtained with new NIK antibody because initial antibody is no longer available. In two cell lines (BEAS2B and H358) the new antibody revealed better NIK expression than it was shown with original antibody. This observation does not affect the conclusions of the manuscript that some melanoma cell lines exhibit a higher-level NIK protein and a higher level of NIK protein associated with IKK than normal human epidermal melanocytes (NHEMs).

**NIK expression in various cell lines.** Whole-cell extracts were harvested in RIPA buffer and 40 ug of total protein for each cell line were loaded for Western blot. After proteins were transferred to nitrocellulose membrane, the membrane was cut between 50 KD and 75 KD. The upper part was blotted with anti-NIK antibody (Cell Signaling Technology, Cat. # 4994) and signal was detected with Enhanced Chemiluminescence (ECL) method. The lower part of the blot was developed with anti-β-actin antibody (Invitrogen, Cat. # MA5-15739) and signal was detected with fluorescence method (LI-COR). Wild type mouse mammary fibroblast (NIK WT MEF) and NIK knock-out mouse mammary fibroblast (NIK WT MEF) were used as positive and negative controls for the NIK antibody.

**Figure:**